# Cross-Flow Microfiltration of Glycerol Fermentation Broths with *Citrobacter freundii*

**DOI:** 10.3390/membranes10040067

**Published:** 2020-04-08

**Authors:** Wirginia Tomczak, Marek Gryta

**Affiliations:** Faculty of Chemical Technology and Engineering, West Pomeranian University of Technology in Szczecin, ul. Pułaskiego 10, 70-322 Szczecin, Poland

**Keywords:** ceramic membrane, chemical cleaning, cross-flow microfiltration, fermentation broth, fouling analysis

## Abstract

This paper reports the study of the cross-flow microfiltration (MF) of glycerol fermentation broths with *Citrobacter freundii* bacteria. A single channel tubular ceramic membrane with a nominal pore size of 0.14 µm was used. It has been demonstrated that the MF ceramic membrane has been successfully applied to bacteria cell removal and to effectively eliminate colloidal particles from glycerol fermentation broths. However, due to fouling, the significant reduction of the MF performance has been demonstrated. In order to investigate the impact of transmembrane pressure (TMP) and feed flow rate (Q) on MF performance, 24 experiments have been performed. The highest steady state permeate flux (138.97 dm^3^/m^2^h) was achieved for 0.12 MPa and 1000 dm^3^/h. Fouling analysis has been studied based on the resistance-in series model. It has been found that the percentage of irreversible fouling resistance during the MF increases with increasing TMP and Q. The permeate flux regeneration has been achieved by membrane cleaning with 3 wt % NaOH and 3 wt % H_3_PO_4_ at 45 °C. The results of this study are expected to be useful in industrially employing the MF process as the first step of glycerol fermentation broth purification.

## 1. Introduction

Microfiltration (MF) represents an alternative to conventional filtration processes and recently it is the mainstream separation technique used to treat suspensions. It is a pressure-driven process mainly applied for the removal of particles in the range of 0.1–10 µm from a liquid. Hence, during microfiltration bacteria and particles that contribute to the suspension turbidity can be successfully retained by a membrane. 

Table A1, Appendix A summaries literature review related to the cross-flow MF of bacterial suspensions by using ceramic membranes. It indicates that microfiltration process is becoming increasingly attractive in the biotechnology industry in order to obtain a clarified filtrate from various bacterial products such as skim milk [1,2,3,4,5,6], skimmed colostrum [7], gum arabic suspension [8], cell suspension [9,10,11,12], fermentation soy sauce [13,14], and fermentation broths [15,16,17,18,19,20,21,22,23,24,25]. It has to be pointed out that with regard to complex media such as fermentation broths, microfiltration is proposed as a pre-treatment stage for the final separation by nanofiltration (NF) or reverse osmosis (RO) processes with spiral-wound modules [26].

Ceramic membranes have potential application in the clarification of biological suspensions and in recent years they have gained an important role in industrial processes. It is worth mentioning that for separation of fermentation broths, ceramic membranes are much more commonly used than other types of membranes. This is due to the fact that they have many advantages. It has been well documented that inorganic membranes offer: (i) chemical and thermal stability, (ii) bacteria resistance, (iii) high abrasion resistance, (iv) high flues and high separation efficiency, (v) high porosity and narrow pore distribution, and (vi) long service life [27,28,29,30,31,32]. Microfiltration membranes used for the clarification of fermentation broths usually have a microporous structure with a mean diameter of pores in the range of 0.10–1.40 µm and they are generally manufactured from compounds such as Al_2_O_3_, TiO_2_, and ZrO_2_ (Table A1).

It is well known that the significant issues that limit the industrial application of cross-flow microfiltration of biological suspensions are concentration polarization and membrane fouling. The general effect of these complex phenomenon is the permeate flux decline during an operation. Indeed, it leads to the reduction of the productivity of the system and makes membrane cleaning a necessity. This causes in increasing operational costs and reduces a membrane’s lifetime [33,34,35,36]. Based on literature review, it can be analyzed that flux decline during MF of biological suspensions is affected by a great number of factors, such as: (i) process parameters (transmembrane pressure, feed flow rate, and temperature) [1,2,4,6,12,16,18,19,20,21,22,23,25,37,38], (ii) membrane properties (pore size and its distribution, hydrophilicity/hydrophobicity character) [1,3,6,14,21,25,37,39], (iii) feed solution properties (nature, bacterial cell mass, particle size, and pH) [11,12,21,22,38], and (iv) interaction between foulants and membranes [3,22]. It should be mentioned that these interactions are often unknown or not understood at the fundamental level [40]. Therefore, better understanding of overall fouling is the prime objective to develop membrane processes and increase membrane flux [25,35]. Although MF research has gained immense importance over the past decade [41], based on literature review, studies focusing on the fouling importance during microfiltration of fermentation broths in cross-slow systems with ceramic membranes are very limited (Table A1).

Resistance analysis is a very effective method in order to the determine the phenomena leading to permeate flux decline during filtration processes [25]. Thus, in several previous studies [2,8,11,13,14,16,19,24,25,37,42,43,44,45] the resistance-in series model has been successfully applied to analyze the reduction of permeate flux during cross-flow MF of various microbial media. This model classified fouling resistance into resistances of: membrane, polarization, adsorption, as well as cake built on the membrane surface. For instance, Carrère et al. [43] have indicated that during MF of lactic acid fermentation broths the resistances due to adsorption and solute concentration polarization are dominated. In turn, in [44], it has been demonstrated that during MF of *Bacillus subtilis* fermentation broths the cake resistance formed by bacteria cells and extracellular polymeric substances (EPS) play an important role in determining the overall resistance during the process. 

In principle, the flux regeneration is one of the most important criteria from an economical point of view in membrane filtration processes [46]. Therefore, chemical cleaning is an integral part of membrane processes since it offers the possibility to remove hydraulically irreversible foulants and hence reduce membrane fouling and provide high process effectiveness [47,48,49]. A number of studies in the literature have shown that five types of chemical cleaning reagents are employed: bases, acids, disinfectants, surfactants, and chelates. Commercial cleaning products (like Ultrasil) are often mixtures of these compounds, but the composition is unknown in the public domain [50]. It has to be pointed out that choosing the best chemical products or its combinations requires knowledge of the feed composition, membrane material, and precipitated layers on the membrane surface [40,51]. Thus, effectiveness of chemical cleaning depends on interactions between used chemical agents and macromolecules in membrane fouling layers. For cleaning membranes fouled by bacterial suspensions, the most commonly used are: NaOH, HNO_3_, C_6_H_8_O_7_, and NaClO (Table A1). However, combining alkaline and acid cleaning is often required. For instance, in the microfiltration of a fermentation broth with *Actinobacillus succinogenes* ATCC 55618 Thuy and Boontawan [24] demonstrated that combining caustic (1 wt % NaOH) and acid (1.5 wt % H_3_PO_4_) cleans is an effective method of cleaning a ceramic membrane. In turn, in [25], it has been found that ceramic membrane fouled by components of cellulase fermentation broth can be effectively cleaned by 1 wt % NaOH and 0.1 mol/L citric acid (C₂H₂O₄) solution.

To date, it has been documented that *Citrobacter freundii* are widely cultivated microorganisms for the biotechnological production of 1,3-propanediol through glycerol fermentation process [52,53,54,55,56]. Although the literature widely reports the use of MF for various microbial fluids purification (Table A1), to the best of the author’s knowledge, there are still significant gaps in studies focusing on the cross-flow microfiltration of glycerol fermentation broths with *Citrobacter freundii* bacteria using ceramic membranes. In response to the state of the existing literature, the overall aim of the experiments was to apply the MF process with the ceramic membrane in the cross-flow system to the treatment of glycerol fermentation broths with *Citrobacter freundii* and evaluate the influence of the operating conditions: transmembrane pressure and feed flow rate on the MF process performance. In addition, for each feed flow rate the critical flux has been determined. Furthermore, the study focuses on the fouling analysis bases on the resistance-in series model. Finally, it presents an approach to effective method of membrane cleaning.

## 2. Materials and Methods 

**Figure 1:**
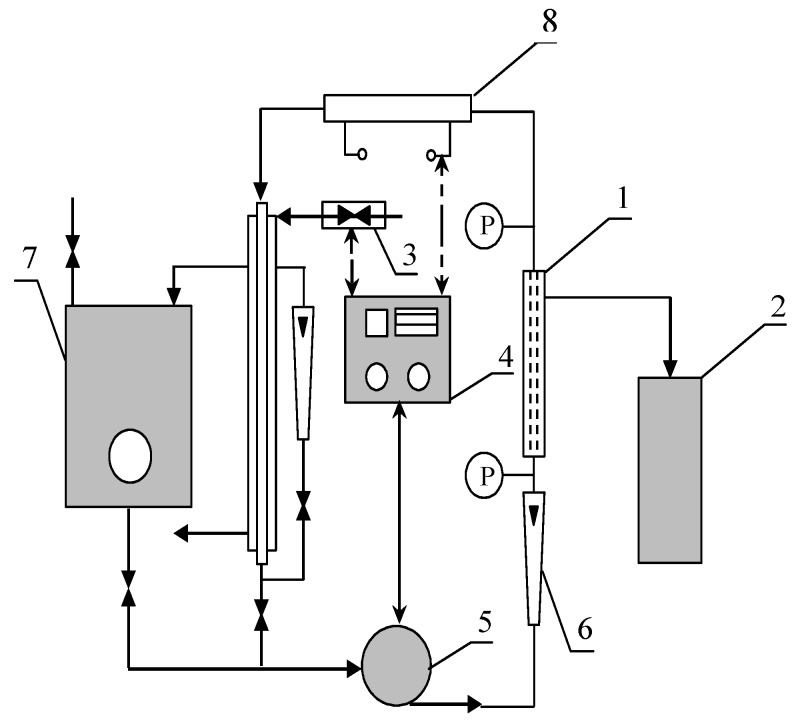
Experimental set-up of the cross-flow microfiltration (MF) unit. 1—MF module, 2—measuring cylinder, 3—heat exchanger, 4—controller of temperature and flow rate, 5—pump, 6—rotameter, 7—feed tank, 8—heater, and P—manometer.

Cross-flow microfiltration experiments were carried out in a pilot scale system (INTERMASZ, Września, Poland) represented in Figure 1. The experimental apparatus is composed of four major parts: membrane module for cross-flow filtration made of 316 stainless steel (AISI 316 L), feed tank, controller of temperature and flow rate, and circulation pump.

Transmembrane pressures (TMP) were calculated as follows:

(1) $$\mathrm{TMP}=\frac{P_{\mathrm{IN}}+P_{\mathrm{OUT}}}{2_{}}-P_{P}$$

where P_IN_ is inlet pressure, P_OUT_ is outlet pressure, and P_p_ is pressure on the filtrate side of the membrane.

Before each experiment the initial pure water flux J_0_ was measured. Distilled water fluxes were measured with the permeate side open for 10 min under controlled temperature equal to 30 °C, constant transmembrane pressure in the range from 0.02 to 0.12 MPa and volumetric flow rate equal to 500 dm^3^/h which corresponds to the cross-flow velocity 5.46 m/s and Reynolds number 30505.

The experiments have been performed in a conventional cross-flow microfiltration unit. The single channel tubular ceramic membrane (TAMI Industries, Lyon, France) used had a nominal pore size of 0.14 µm, internal diameter 5.6 mm and length 220 mm. The useful membrane surface S was equal to 3868 mm^2^. According to the manufacturer, the selective layer was zirconium bound on a titanium oxide support.

Microfiltration experiments were performed with fresh glycerol fermentation broths with cultures of *Citrobacter freundii* inoculated under sterile conditions in a bioreactor (bacteria culture volume comprised 5% of the total reactor volume). The medium for the cultivation phase contained the following components (g/L): glycerol (20), peptone K (2.5), meat extract (1.5), yeast extract (2.0), K_2_HPO_4_ (3.4), MgSO_4_*·*7H_2_O (0.4), CaCl_2_ (0.08), CoCl_2_ (0.002), KH_2_PO_4_ (1.3), and (NH_4_)_2_SO_4_ (2.0). A two days fermentation process was performed under agitation at 150 *±* 5 rpm, the incubation temperature was equal to 30 °C. The pH value was maintained at 7.0 by automatic additions of 5 M solution of sodium hydroxide (NaOH).

All cross-flow microfiltration experiments were carried out for 250 min at constant temperature equal to 30 °C. The feed volume was 2 L. 

The permeate flux J during the microfiltration of fermentation broth was determined by measuring the permeate cumulative volume dV in defined time intervals dt:

(2) $$J=\frac{1}{S}\frac{\mathrm{dV}}{{\mathrm{dt}}_{}}$$

where S is the total active membrane area (m^2^).

MF experiments have been performed out under constant transmembrane pressure as it is recommended for working with suspensions when steady state permeate fluxes can be achieved [57]. In order to investigate the impact of transmembrane pressure and feed flow rate on microfiltration performance 24 experiments have been performed. MF processes were carried out at six different transmembrane pressures from 0.02 to 0.12 MPa and at four different volumetric feed flows from 250 to 1000 dm^3^/h which correspond to the cross-flow velocity in the range from 2.82 to 11.28 m/s and the Reynolds number between 15,252 and 61,010.

In order to regain the membrane permeability after each microfiltration experiment cleaning of the MF system was carried out. The membrane was first rinsed with water at 30 °C for 10 min. It was then cleaned with a 3 wt % solution of sodium hydroxide (NaOH) for 60 min at 45 °C, followed by rinsing with water for 10 min. Lastly, it was cleaned with a 3 wt % solution of phosphoric acid (H_3_PO_4_) for 60 min at 45 °C and rinsed once more with water at 30 °C for 10 min (Table 1). During all steps feed flow rate was equal to 500 dm^3^/h and permeate outlet was closed (TMP = 0). To evaluate the results of the cleaning procedure after each step the permeate flux for distilled water was measured.

The proposed method of membrane cleaning was considered effective since after each MF process the original membrane permeability was regained. 

Flux decline during MF process was demonstrated by a drop in relative flux, J_r_, defined as the ratio between the actual permeate flux rate and permeate flux determined for a new (clean) membrane:

(3) $$J_{r}=\frac{J}{J_{0}}$$

where J_0_ is the pure water flux of a new (clean) membrane.

The hydraulic resistance of the clean membrane R_m_ was obtained from the flux measurement of pure water under various TMP (in the range between 0.02 and 0.12 MPa) according to the Darcy’s law:

(4) $$R_{m}=\frac{\mathrm{TMP}}{{\;\mu }_{W}J_{0}}$$

where TMP refers to the transmembrane pressure (Pa), µ_w_ is the viscosity of water at temperature 30 °C (Pa·s).

The total membrane resistance R_T_ was calculated at steady state conditions after 250 min of each filtration experiment, using following equation:

(5) $$R_{T}=\frac{\mathrm{TMP}}{{\;\mu }_{P}J_{S}}$$

where µ_p_ is the viscosity of the permeate solution at 30 °C (Pa·s) and J_s_ is the steady state permeate flux (m/s).

In the present study the total membrane resistance R_T_ was assumed to be the sum of hydraulic resistance of the clean membrane R_m_ and fouling resistance R_f_ which includes reversible fouling resistance R_rev_ and irreversible fouling resistance R_irr_:

R_T_ = R_m_ + R_rev_ + R_irr_ (6)

Reversible fouling resistance R_rev_ is due to concentration polarization and it can be removed by rinsing with water after the filtration run, whereas irreversible fouling resistance R_irr_ is the result of membrane pore blocking and adsorption of broths components on the membrane surface and/or it pores and it requires chemical cleaning [11]. Irreversible and reversible fouling resistances have been determined according to the following equations:

(7) $$R_{\mathrm{irr}}=R_{T}-R_{m}-\frac{\mathrm{TMP}}{{\;\mu }_{P}J_{W}}$$

R_rev_=R_T_ − R_m_ − R_irr_ (8)

where J_w_ is the pure water flux after membrane rinsing.

Physio-chemical properties of the fermentation broth such as: (i) composition, (ii) turbidity, (iii) pH, (iv) dynamic viscosity, (v) number of bacteria, and (vi) total wet biomass were measured before and after the microfiltration experiments. The concentrations of glycerol, 1,3-PD and the organic acids were determined by high performance liquid chromatography HPLC using a UlitiMate 3000 (Thermo Fisher Scientific, Germering, Germany) with refractometer detector R1-101 Shodex (Showa Denko America, New York, NY, USA) and column Aminex HPX-87H (BIO RAD, Berkeley, CA, USA) with HyperREZ XP H+ Guard (Thermo Scientific, Waltham, MA, USA), through which a 5 mM H_2_SO_4_ solution at a flow rate 0.6 mL/min. Determination of anions and cations in the tested solutions was carried out using an 850 Professional IC ion chromatograph (Herisau Metrohm AG, Herisau, Switzerland) with column Hamilton PRP-X300; 250 × 4.1 mm (Hamilton Company, Berkeley, CA, USA) using as the mobile phase 0.5 mM H_2_SO_4_ at a flow rate 1.0 mL/min, operating at temperature equal to 30 °C. Turbidity (NTU scale) of feed and permeate samples was analyzed using a HACH (Hach Company, Loveland, CO, USA) turbidimeter (2100ANIS). The values of pH were measured by using the multifunctional ULTRAMETER 6P meter (Myron L Company, Carlsbad, CA, USA). Viscosity was determined making measurements in a viscometer (BROOKFIELD DV-II + Pro) with UL Adapter (BROOKFIELD ENGINEERING LABORATORIES, Middleboro, MA, USA). The number of CFU bacteria in fermentation broth was determined by inoculation on plates and counted after 24 h of incubation in a Nuve NE055 incubator at 30 °C using a POL-EKO LKB 2002 colony counter (POL-EKO-APARATURA, Wodzisław Śląski, Poland). A biomass concentration was determined by measuring the weight of wet biomass present in 0.1 L of broth (6000 rpm, centrifuge MPW-350R, Med-Instruments, Warszawa, Poland).

Clarity C_r_ was determined by measurements of turbidity ratio between feed and permeate samples in defined periods of time [8]:

(9) $$C_{r}=\left(1-\frac{\tau_{P}}{{\;\tau }_{f}}\right)\times 100\%$$

where τ_p_ and τ_f_ are the turbidity of permeate and feed, respectively.

## 3. Results and Discussion

### 3.1. Fermentation Broths

The fermentation broths that were a fed to the microfiltration contained the following components: 1,3-propanediol (1,3-PD), lactic acid, acetic acid, Cl^−^, NO_3_^−^, PO_4_^3−^, SO_4_^2−^, Na^+^, NH_4_^+^, K^+^, Ca^2+^, and Mg^2+^ (Table 2). It should be mentioned that since compounds dissolved in fermentation broths were not rejected by the MF membrane, the solute concentrations in the permeate were the same as to those determined in the feed.

Table 3 shows the physicochemical characteristic of glycerol fermentation broths with *Citrobacter freundii* bacteria. The turbidity of the feed was in the range between 1700 and 2100 Nephelometric Turbidity Unit (NTU). pH was equal to 7 which is the suitable pH for the *Citrobacter freundii* growth. Dynamic viscosity of fermentation broths has been reported as 0.85 × 10^−3^ Pa·s. In turn, number of bacteria in the feed was in the range between 3.55 × 10^7^ and 5.48 × 10^9^ CFU/mL. The same order of magnitude of CFU/mL in various bacterial suspensions clarified by cross-flow MF systems with ceramic membranes has been reported in previous studies [5,7,11,16,19,20,23] (Table A1). In the fermentation broths, a sediment has been observed and the total wet biomass has been determined in the range 5.06 and 10.08 g/dm^3^.

### 3.2. The Efficiency of Microfiltration Process 

The rejection of bacterial cells and particles causing the turbidity of fermentation broths was crucial in order to verify the microfiltration efficient. Therefore, the efficiency of the process was estimated based on turbidity measurements, permeate clarity, and determining the count of bacteria in the obtained permeate.

**Figure 2:**
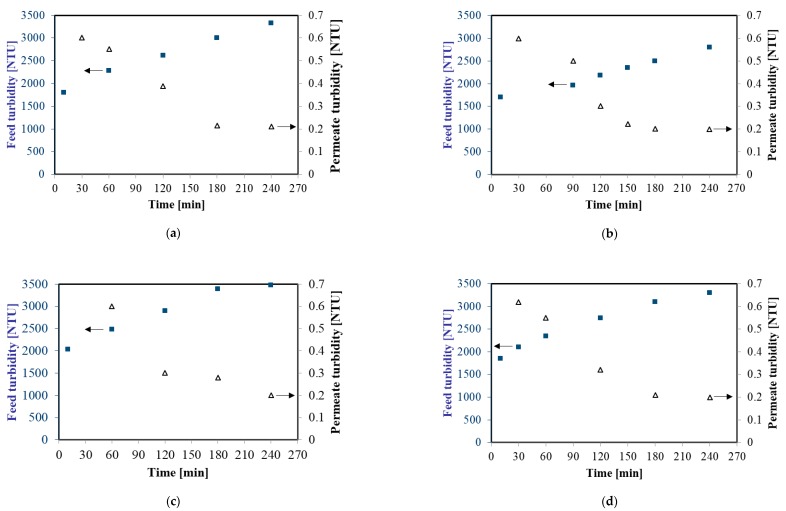
Changes of feed and permeate turbidity during cross-flow MF process, Transmembrane Pressures (TMP) = 0.08 MPa. (**a**) 250 dm^3^/h; (**b**) 500 dm^3^/h; (**c**) 750 dm^3^/h; and (**d**) 1000 dm^3^/h.

Figure 2 shows changes of feed and permeate turbidity during MF process at TMP equal to 0.08 MPa and four selected feed flow rates (from 250 to 1000 dm^3^/h). The initial turbidity of the fermentation broths was in the range 1700–2100 NTU and it systematically increased to 2800–3500 NTU, as a result of broths thickening. For instance, for 750 dm^3^/h during the first hour of the experiment, increasing of the feed turbidity from 2034 to 2478 NTU has been observed. Then, the turbidity was equal to 2901 NTU, 3389 NTU, and 3478 NTU in the second, third, and fourth hour of the experiment, respectively. In contrast, the turbidity of treated samples decreased significantly over the MF time. After one hour of each experiment, the turbidity of permeate was equal to about 0.6 NTU whereas after four hours it has been equal to 0.2 NTU. This observation indicated that during the MF process a fouling layer was formed on the membrane surface which led to enhance its separating effectiveness. An important point which should be noted is that the results obtained from measurements of the feed and permeate turbidity demonstrate a significant degree of colloidal particle retention by the used MF membrane. Importantly, it demonstrates that applying MF membranes with a nominal pore size equal to 0.14 µm allowed one to obtain a permeate of the quality required for NF or RO processes with spiral-wound modules.

Obtained permeate clarity (C_s_) (Equation 9) throughout all experiments was higher than 99.9%. It indicates that almost all insoluble fine particles were removed from the fermentation broths. It has to be pointed out that C_s_ obtained in the present work is higher than C_s_ reported in [8]. Bechervaise et al. [8] obtained C_s_ between 78.9% and 88.3% during removal of thermophilic spores, from gum Arabic streams, by a ceramic membrane with a pore size equal to 0.80 µm. Moreover, turbidity of the permeate obtained in the present work was slightly lower than those reported in previous studies [13,14,25] where purification of various solutions by microfiltration membranes has been studied. For example, in [13] it has been demonstrated that during the clarification of raw soy sauce (feed turbidity: 48 NTU) by a ceramic membrane with the pore size diameter of 0.20 µm allowed to obtain the turbidity of the final product equal to 0.41 NTU and the removal ratio 99.1%. In turn, Yang et al. [25] reported turbidity of permeate equal to 0.81; 0.52 and 0.56 NTU, respectively, during purification of cellulase fermentation broth (feed turbidity: 646 NTU) via ceramic MF membranes with three different pore sizes: 0.05; 0.20, and 0.50 µm.

Importantly, the membrane performance in terms of sterility was satisfactory. It is related to the fact that no bacteria were detected in permeate samples obtained during all microfiltration experiments. It indicates complete removal of *Citrobacter freundii* bacteria cells from fermentation broths. These observations are related to the large difference in size between the bacterial cells and the membrane pores. Results obtained in this work clarifies that the MF ceramic membrane with a nominal pore size of 0.14 µm has been successfully applied in order to bacteria cells removal and effectively eliminates of colloidal particles from glycerol fermentation broths. Hence, MF can be industrially employed as the first step of glycerol fermentation broth purification.

### 3.3. Effect of the Operating Pressure

**Figure 3:**
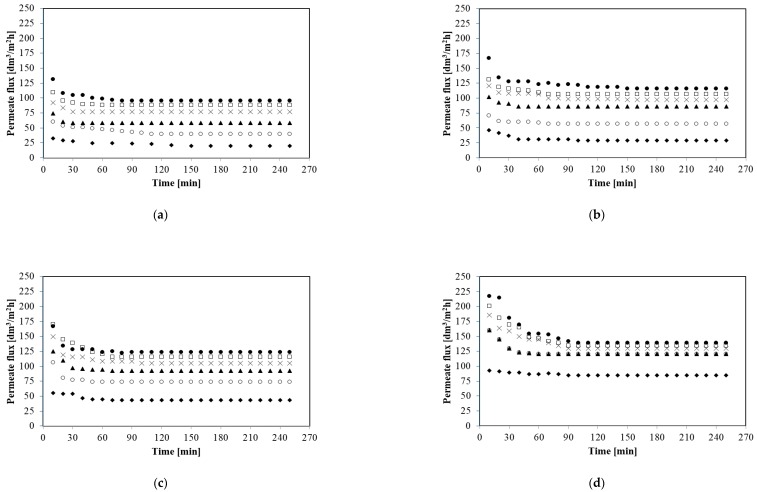
Effect of transmembrane pressure on the permeate flux. (**a**) 250 dm^3^/h; (**b**) 500 dm^3^/h; (**c**) 750 dm^3^/h; and (**d**) 1000 dm^3^/h. TMP [MPa]: ◆ 0.02, ○ 0.04, ▲ 0.06, × 0.08, □ 0.10, and ● 0.12.

It is well known that transmembrane pressure is one of the most important parameters affecting microfiltration process performance. Therefore, the initial aim of microfiltration studies was to examine the effect of TMP on the permeate flux. Hence, MF processes have been carried out under constant transmembrane pressure in the range from 0.02 to 0.12 MPa for four different feed flow rates: 250; 500; 750, and 1000 dm^3^/h. Figure 3 shows the variation in the permeate flux during the microfiltration processes. 

As it has been demonstrated (Figure 3) the reduction of the microfiltration performance is evident for all analyzed experimental conditions. Moreover, the profiles of permeate flux decline were similar for all applied values of transmembrane pressure and feed flow rate. The results revealed that the permeate flux decreased significantly within the initial phase of 10–20 min and reached a steady state after about 60 min. For example during the first 20 min of the process for the feed flow rate 250 dm^3^/h and TMP 0.02 MPa the permeate flux decreased from 152.86 to 29.43 dm^3^/m^2^h and after 60 min the flux was stable and equal to 20.07 dm^3^/m^2^h. In turn, for the feed flow rate 750 dm^3^/h and TMP 0.08 MPa during the first 20 min the decline of the permeate from 583.67 to 115.81 dm^3^/m^2^h has been noted. Finally, the permeate flux was constant and equal to 105 dm^3^/m^2^h. 

Decline in the permeate flux with time of the MF process was related to the fact that during the filtration process membranes tend to be affected by components presented in a feed stream [58]. This decline can be due to the inevitable phenomena of either concentration polarization or formation of a cake layer by bacteria cells and other components of fermentation broths (fouling). Concentration polarization is a natural consequence of the membrane selectivity and it leads to reversible build-up of particles or dissolved solutes in a layer adjacent to the membrane surface and thus it can decrease the permeate flux [57]. In turn, fouling is a very complex physicochemical phenomenon [59]. It is related to the fact that it is associated with several possible causes such as cake or gel formation and deposition on the membrane surface, or the plugging of membrane pores feed stream components [60]. These phenomena provide an additional increasing resistance to MF process thus the permeate flux decreases with time.

Analyzing the data shown in Figure 3, it can be concluded that the highest steady state flux values were obtained for the highest TMP at all feed flow rates. For example for 250 dm^3^/h and TMP 0.02 MPa the steady state permeate flux was equal to 20.07 dm^3^/m^2^h whereas for TMP 0.12 MPa it was equal to 95.73 dm^3^/m^2^h. In turn, for 1000 dm^3^/h a six-fold increase of TMP (from 0.02 to 0.12 MPa) led to an increase steady state permeate flux from 84.93 to 138.97 dm^3^/m^2^h. It indicates that TMP has the important impact on MF process performance. It has to be pointed out that for all feed flow rates tested, the increase in process efficiency was particularly significant when increasing of pressure from 0.02 to 0.04 MPa has been applied.

It can be expected that steady state permeate flux increases with the TMP increase, as it is the driving force in MF process [61] and enhanced driving force for solvent flux was bigger than the membrane fouling resistance [62].

Results obtained in the present work are comparable with previous studies [2,4,12,18,21,25] which demonstrated that in general increasing TMP may lead to increase of permeate flux during MF of bacterial suspensions. However, in [12] it has been found that during MF of cyanobacterial strain *Arthrospira* sp the permeate flux is pressure dependent but only up to TMP equal 0.20 MPa. Likewise, Milcent and Carrère [21] have shown that during MF of lactic acid fermentation broths the steady state permeate flux is transmembrane pressure independent above 0.035 MPa. Moreover, Yang et al. [25] have investigated the effect of TMP (in the range from 0.05 to 0.20 MPa) on the steady state permeate flux during MF of cellulase fermentation and they observed the positive effect of TMP increasing only up to 0.10 MPa. Furthermore, results obtained in the present work are inconsistent with those obtained in [63] where it has been observed that during MF of fermentation broth with *Bacillus subtilis*, permeate flux is almost independent of the pressure applied.

As a conclusion, for each feed flow rate applied the highest values of permeate flux have been achieved at the highest operating pressure equal to 0.12 MPa. In turn, the lowest MF performance was observed during the run at TMP equal to 0.02 MPa. 

### 3.4. Effect of the Feed Flow Rate

Hydrodynamic conditions are key factors affecting the membrane performance. It is related to the fact that changing the hydrodynamic conditions in the membrane system leads to different deposit layer properties. Hence, the feed flow rate plays an important role in the enhancement of permeate flux during the microfiltration process. It is related to the fact that it affects the mass transport of particles and it leads to decrease the cake layer thickness on the membrane surface [64]. Therefore, it may be possible to increase MF performance by increasing the local shear rates near the membrane surface. On the other hand, a higher cross-flow velocity of a feed solution leads to increase energy demand which effects on economic aspects of the process [4,34,65]. However, according to Streit et al. [23], for the same operational conditions such as transmembrane pressure, increasing the feed cross-flow velocity may reduce MF process time.

**Figure 4:**
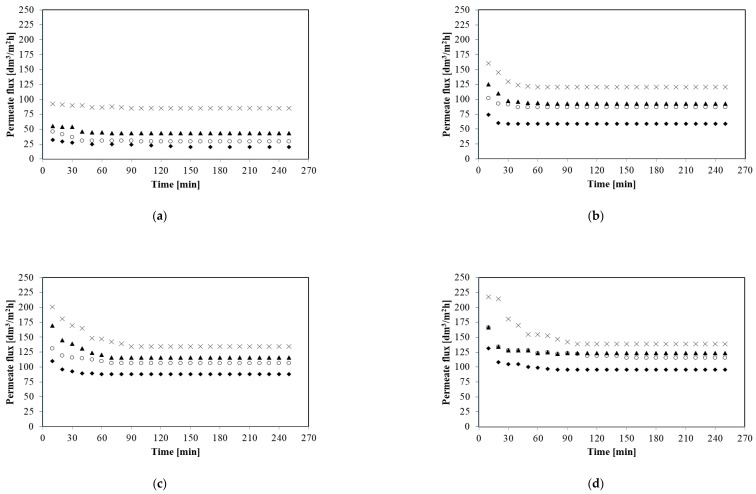
Effect of feed flow rate on the permeate flux. (**a**) 0.02 MPa; (**b**) 0.06 MPa; (**c**) 0.10 MPa; and (**d**) 0.12 MPa. Q [dm^3^/h]: ◆ 250, ○ 500, ▲ 750, × 1000.

In the present study, all of the MF experiments have been carried out under the turbulent feed flow, as the Reynolds number was in the range between 15,252 and 61,010 (Table 1). Figure 4 shows the impact of the feed flow rate (from 250 to 1000 dm^3^/h) on the permeate flux during MF of glycerol fermentation broths with *Citrobacter freundii* bacteria for selected constant transmembrane pressures from 0.02 to 0.12 MPa. It is clearly observed that the feed flow rate has the positive effect on the permeate flux. It has been demonstrated that for TMP equal to 0.06 MPa increase of the feed flow rate (from 250 to 500 dm^3^/h) led to the significant steady state permeate flux improvement, from 58.68 to 86.47 dm^3^/m^2^h, whereas another increase of the feed flow rate up to 1000 dm^3^/h enhanced steady state permeate flux up to 120.44 dm^3^/m^2^h. Similar MF performance enhancement by increasing the feed flow rate has been noted under all applied TMPs. For instance, for TMP equal to 0.10 MPa four-fold increasing of the feed flow rate (from 250 to 1000 dm^3^/h) resulted in increase of the steady state permeate flux from 88.01 to 134.34 dm^3^/m^2^h.

This positive effect of feed flow rate on the permeate flux can be due to the enhanced hydrodynamic effects at the membrane surface. Increase of turbulence may lead to bringing back the particles to the feed solution, decreasing the cake thickness built on the membrane surface and thereby decreasing the concentration polarization and fouling phenomenon [12,61,66]. Moreover, according to Hwang et al. [67] increasing the cross-flow velocity may result in lower average cake porosity and thus enhancement of permeate flux.

Increasing the permeate flux with increasing of the feed flow rate is consistent with overall results presented in previous studies [2,12,23,25] where MF of various bacterial suspensions has been investigated. For example Fritsch and Moraru [2] have demonstrated that increasing the feed flow rate from 5 to 7 m/s led to an almost 10-fold increase in permeate flux (from 4.2 to 40.5 dm^3^/m^2^h). However, in [12] it has been observed that for MF of cyanobacterial strain *Arthrospira* sp., the enhancement in the permeate flux was significant with increasing the cross-flow velocity only below a value of 0.035 m/s.

Summarizing, in the present study, for each of TMP applied (from 0.02 to 0.12 MPa), the highest values of the steady state permeate flux have been obtained at the highest feed flow rate equal to 1000 dm^3^/h (11.28 m/s). By contrast, the lowest MF performance has been noted at the lowest feed flow rate 250 dm^3^/h (2.82 m/s).

### 3.5. Identification of the Critical Fluxes

Membrane fouling is related to the concept of the critical flux. The critical flux is defined as the flux below which a decline of permeate flux with time does not occur [33]. Hence, the concept of the critical flux is important in guiding the operation of microfiltration process [68]. Thereby, it can be applied in order to reduce the flux decline during filtration process and maintain maximum membrane productivity.

**Figure 5:**
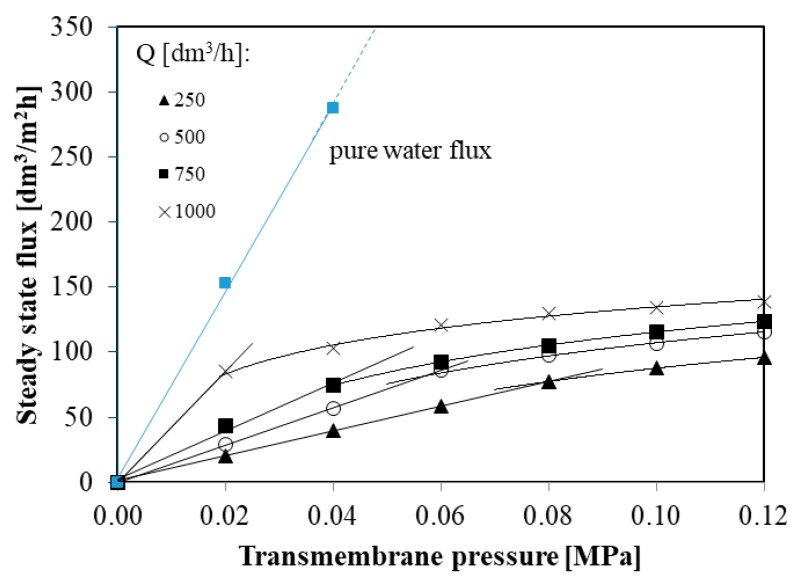
The steady state permeate flux in function of transmembrane pressure at different feed flow rates.

Figure 5 shows steady state permeate flux during the MF process of glycerol fermentation broths in function of transmembrane pressure at different feed flow rates (from 250 to 1000 dm^3^/h).

It is worth noting that for each feed flow rate (from 250 to 1000 dm^3^/h) the steady state permeate flux depends linearly on the transmembrane pressure until the critical flux J_crit_ is reached. Above J_crit_ permeate flux increases more slowly in function of TMP. Since the relation between permeate flux and TMP relationship is below that of the pure water (Figure 5), the critical fluxes obtained in the present study correspond to the weak form of critical flux which has been defined in [33,57]. Weak form of the critical flux has also been reported in several previous studies [17,69,70].

In the present study, critical fluxes were found to be: 77, 86, 74, and 85 dm^3^/m^2^h for feed flow rates 250, 500, 750, and 1000 dm^3^/h, respectively. Although, in previous studies [22,71,72] the influence of the cross flow velocity on the critical flux was noted, in this work no such effect has been observed. This difference may be due to the fact that in this study the MF processes were carried out for turbulent flows characterized by very large Reynolds numbers (from 15,252 to 61,010).

### 3.6. Fouling Analysis

**Figure 6:**
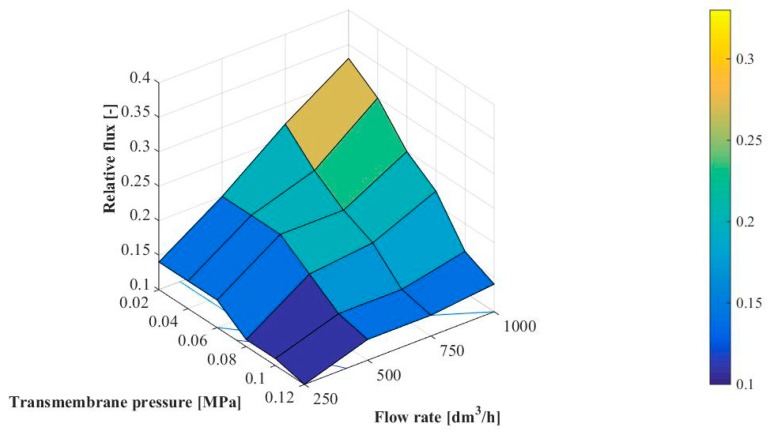
Effect of transmembrane pressure and feed flow rate on the relative flux.

As explained previously, the permeate flux during MF process of glycerol fermentation broths with *Citrobacter freundii* bacteria is strongly affected by transmembrane pressure and feed flow rate. In the present study, the relative flux defined by Equation 4 as the fouling indicator has been applied. Figure 6 shows the effects of TMP and Q on the relative flux J_r_.

The experimental results showed that at the steady state conditions the relative flux was in the range between 0.10 and 0.33. The lowest value of J_r_ has been obtained for the highest TMP (0.12 MPa) and the lowest Q (250 dm^3^/h). According to Hwang et al. [67] and Balcıoğlu and Gönder [73], as the TMP increases, more pollutants accumulate on the membrane surface and it leads to more compact cake or a more compact skin layer as well as membrane pore clogging, due to the increase in driving force. In contrast, the highest value of J_r_ for the lowest transmembrane pressure (0.02 MPa) and the highest feed flow rate (1000 dm^3^/h) has been noted.

Results obtained in the present study demonstrate that for each performed experiment drop of the permeate flux was significant. It has to be pointed out that the identification of flux decline mechanism is very significant for MF processes [74]. Therefore, in order to improve the knowledge on the reduction of permeate flux during the MF of glycerol fermentation broths with *Citrobacter freundii* the resistance-in series model has been applied. The hydraulic resistance of the clean membrane R_m_ was equal to 5.94 × 10^11^ m^−1^. The same R_m_ order of magnitude has been obtained in several previous studies [2,9,11,14,16,21,25] where the MF of microbial suspensions has been examined (Table A1).

**Figure 7:**
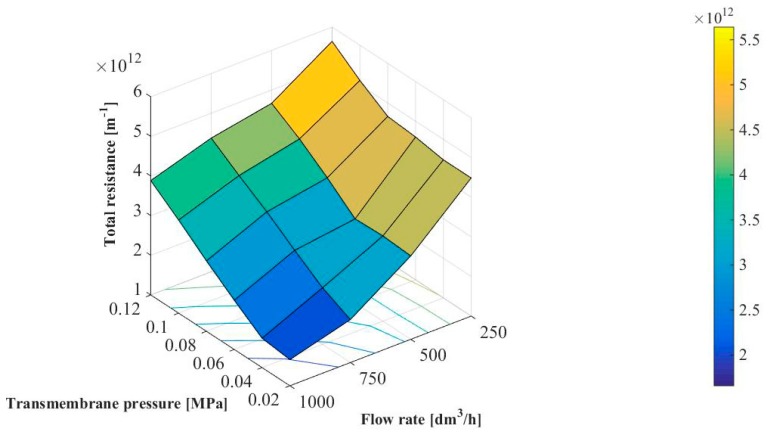
Effect of transmembrane pressure and feed flow rate on the total hydraulic resistance.

Figure 7 shows the effect of transmembrane pressure and feed flow rate on the total hydraulic resistance. It has been demonstrated that values of R_T_ were in the range between 1.66 × 10^12^ and 5.64 × 10^12^ m^−1^. The same R_T_ order of magnitude has been previously reported in [14,25,37] where the microfiltration of various bacterial media by using ceramic membranes has been investigated. For example, Head and Bird [37] have demonstrated that the total hydraulic resistance during MF of milk (1.0 × 10^4^–1.0 × 10^5^ CFU/mL) with a tubular Al_2_O_3_-membrane (the pore size diameter of 12 µm) was in the range from 1.43 × 10^12^ to 2.62 × 10^12^ m^−1^.

It can be clearly observed that the R_T_ increases with increasing transmembrane pressure. It can be due to the fact that increasing of the driving force may enhance the convective flow of numerous bacteria and colloid particle towards the membrane surface which leads to enhanced concentration polarization and deposition phenomenon [14]. In turn, it has been found that R_T_ decreases with increased the feed flow rate. It confirms the positive effect of the flow turbulence and shearing stress on MF performance. In general, increasing Q could lead to a reduction in the thickness of the diffusion layer and, thus, decreasing the concentration polarization and fouling. 

Increasing the total hydraulic resistance with increasing transmembrane pressure and decreasing feed flow rate has also been reported in [14,17] where microfiltration of bacterial suspensions with ceramic membranes has been investigated.

**Figure 8:**
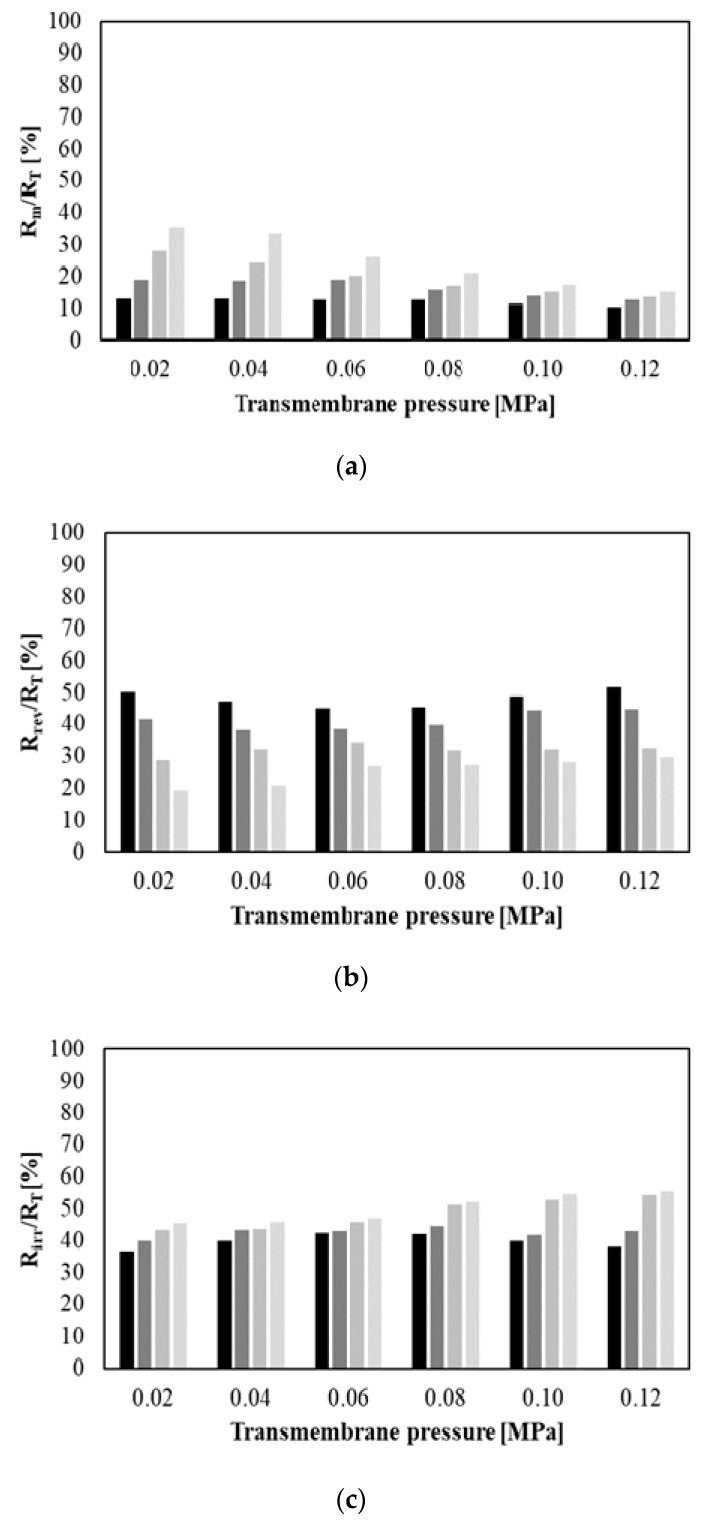
Hydraulic resistance percentages as a function of transmembrane pressure and feed flow rate. (**a**) Membrane resistance; (**b**) reversible fouling resistance; and (**c**) irreversible fouling resistance. Q [dm^3^/h]: 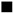 250, 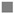 500, 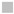 750, 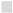 1000.

In turn, Figure 8 shows the relative percentages of hydraulic resistances (membrane R_m_ (a), reversible fouling R_rev_ (b) and irreversible fouling R_irr_ (c)) as a function of TMP for four different feed flow rates (from 250 to 1000 dm^3^/h). The relative percentage of membrane hydraulic resistance was in the range from 10.36% (for 0.12 MPa and 250 dm^3^/h) to 35.22% (0.02 MPa and 1000 dm^3^/h). In general, percentage of R_m_ (values up to 15%) was negligible compared with the R_rev_ and R_irr_ resistances for TMP equal to 0.12 MPa and Q equal to 250 dm^3^/h. This observation indicates that the percentage of membrane hydraulic resistance increases with decreasing of transmembrane pressure and increasing of the feed flow rate. The same observation has been reported in previous studies [14,25]. 

In turn, the percentage of reversible fouling resistance was in the range from 19.36% (for 0.02 MPa and 1000 dm^3^/h) to 50.17% (for 0.02 MPa and 250 dm^3^/h). It has been observed that the percentage of R_rev_ decreases with increasing the feed flow rate. It indicates that reduction of concentration polarization was achieved at the highest Q. The same relationship between reversible fouling resistance and feed flow rate was presented in [14,25].

On the other hand, the percentage of irreversible fouling resistance was noted in the range from 36.78% (for 0.02 MPa and 250 dm^3^/h) to 55.38% (for 0.12 MPa and 1000 dm^3^/h). In general, R_irr_ had a tendency to increase with increasing transmembrane pressure and feed flow rate. It is related to the fact that the increasing of driving force leads to enhance deposition of particles and increase of the fouling layer thickness on the membrane surface [36].

On the basis of the above observations it can be concluded that generally during MF of glycerol fermentation broths the reversible fouling resistance was dominant (percentage ˃ 50%) under the lowest values of TMP (0.02 MPa) and Q (250 dm^3^/h). On the other hand, irreversible fouling resistance was dominant under the highest TMP (from 0.08 to 0.12 MPa) and Q (750 and 1000 dm^3^/h).

## 4. Conclusions

Microfiltration was successfully employed for purification of glycerol fermentation broths. It has been demonstrated that using the ceramic membrane with a nominal pore size equal to 0.14 µm allowed for complete removal of *Citrobacter freundii* bacteria. Moreover, the membrane removed 99.97% of turbidity from the fermentation broths. The results obtained in the present study show that MF performance is strongly affected by transmembrane pressure and feed flow rate. Among all analyzed process conditions the highest steady state permeate flux (138.97 dm^3^/m^2^h) has been obtained for the transmembrane pressure equal to 0.12 MPa and feed flow rate equal to 1000 dm^3^/h. In the present study, for each feed flow rate the weak form of critical flux has been determined. It has been found that the percentage of irreversible fouling resistance increases with increasing transmembrane pressure and feed flow rate. Finally, although permeate decline during MF process was significant, fluxes regeneration has been achieved by membrane cleaning with 3 wt % NaOH and 3 wt % H_3_PO_4_ at 45 °C. The results of this study are expected to be useful in industrially employing the microfiltration process as the first step of glycerol fermentation broth purification.

## Figures and Tables

**Table 1:** Operating conditions for MF process and membrane cleaning.

Step	Q (dm^3^/h)	u (m/s)	Reynolds Number	TMP (MPa)	T (°C)	t (min)	R
Pure water flux	500	5.64	30,505	0.02–0.12	30	10	R_m_
Filtration - fouling	250–1000	2.82–11.28	15,252–61,010	0.02–0.12	30	250	R_T_
Pure water rinsing	500	5.64	30,505	0	30	10	R_irr_; R_rev_
3% NaOH cleaning	500	5.64	30,505	0	45	60	-
Pure water rinsing	500	5.64	30,505	0	30	10	-
3% H_3_PO_4_ rinsing	500	5.64	30,505	0	45	60	-
Pure water rinsing	500	5.64	30,505	0	30	10	-
Pure water flux (cleaned membrane)	500	5.64	30,505	0.02–0.12	30	10	R_m_

**Table 2:** The composition of glycerol fermentation broths with *Citrobacter freundii* bacteria.

**Component**	1,3-PD	lactic acid	acetic acid	Cl^−^	NO_3_^−^	PO_4_^3−^	SO_4_^2−^	Na^+^	NH_4_^+^	K^+^	Ca^2+^	Mg^2+^
**Concentration (g/L)**	9.03–12.73	0.18–0.34	2.16–2.92	0.11–0.15	0.01–0.02	2.09–2.56	1.62–1.83	1.15–1.40	0.56–0.76	1.43–1.58	0.03–0.05	0.03–0.06

**Table 3:** Physicochemical characteristic of glycerol fermentation broths with *Citrobacter freundii* bacteria.

Turbidity (NTU)	pH	Dynamic Viscosity (Pa·s)	Number of Bacteria (CFU/mL)	Total Wet Biomass (g/dm^3^)	Sediment (yes or no)
1700–2100	7.0	0.85 × 10^−3^	3.55 × 10^7^–5.48 × 10^9^	5.06–10.08	yes

## Appendix A

**Table A1:** Summary of the cross-flow MF of bacterial suspensions by using ceramic membranes: experimental features.

Fouling Solution Characteristic			Membrane Characteristic			MF Process			Fouled Membrane Cleaning				Ref.
Solution type	Bacteria	[CFU/mL]	Material	Pore size [µm]	Resistance [m^−1^]	T [°C]	TMP [MPa]	u [m/s]	Cleaning agent	T [°C]	t [min]	u [m/s]	
milk	NI	5.0 × 10^4^–2.0 × 10^5^	ZrO_2_–TiO_2_/TiO_2_; TiO_2_/TiO_2_; multilayer α-alumina	1.40	NI	21; 45	0.05	6.0; 8.0	1% P-3 Ultrasil 25; 1% HNO_3_	75; 50	15	NI	[1]
skim milk	NI	4.1 × 10^3^–1.8 × 10^5^	NI	1.40	3.00 × 10^11^	6 ± 1	0.05– 0.13	5.0– 7.0	20 g/L Ultrasil 25; 5 mL/L HNO_3_	85; 50	50; 15	NI	[2]
skim milk	*Bacillus licheniformis* (FSL strain F4-0073); *Geobacillus* sp. (FSL strain W8-0032)	1.3 × 10^6^–9.6 × 10^6^	NI	1.40; 1.20	NI	6	0.07	4.1	17.5 mL/L Ultrasil 25; 5 mL/L HNO_3_	80; 50	30; 20	NI	[3]
skim milk	*Clostridium tyrobutyricum*; *Bacillus cereus*	NI	Al_2_O_3_	1.00	NI	NI	0.01– 0.14	1.0– 2.0	acid (DIVOS 2); alkaline (DIVOS 124)	NI	NI	NI	[4]
skim milk	NI	1.0 × 10^8^–1.0 × 10^9^	NI	1.40	NI	50	0.10	5.0	Ultrasil 11	NI	NI	NI	[5]
skim milk	*Bacillus anthracis* (Sterne)	1.0 × 10^6^	NI	0.80; 1.40	1.40 × 10^12^	50	0.13	6.2	0.5% NaOH+ 0.5% NaClO; 0.5% HNO_3_	70; 60	30; NI	NI	[6]
skimmed colostrum	*Listeria innocua* ATCC 33090, *Escherichia coli* DSM 30083, *Bacillus subtilis* ATCC 6051	1.8 × 10^9^; 3.0 × 10^8^; 2.0 × 10^7^	NI	1.40; 0.80	NI	30 ± 2	0.07	4.0	NI	NI	NI	NI	[7]
gum arabic suspension	*Bacillus mycoides*	1.0 × 10^5^	Al_2_O_3_	0.80	1.06 × 10^12^	50	0.29	8.5	0.5% NaOH + 200 ppm NaOCl; 0.1% C_6_H_8_O_7_	60	30	11	[8]
cell suspension	*Bacillus cereus* CUETM 98/4	1.0 × 10^6^	ZrTiO_4_	0.45	1.00 × 10^11^–1.00 × 10^12^	15–20	0.08	4.0	0.5% NaOH, 0.5% HNO_3_	55	30	4.0	[9]
cell suspension	*Mycobacterium* M156	NI	Al_2_O_3_	NI	NI	25	0.05– 0.30	NI ^1^	NI	NI	NI	NI	[10]
cell suspension	*Escherichia coli*	2.0 × 10^8^	NI	0.20	4.48 × 10^11^	25	0.15	2.4	10 g/L NaOH; 5 mL/L HNO_3_	80; 60	30	2.4	[11]
cell suspension	*Arthrospira* sp.	NI	kaolinite clay; Al_2_O_3_	1.00 ± 0.39	NI	NI	0.05– 0.35	NI ^2^	1 M NaOH	NI	NI	NI	[12]
fermentation soy sauce	NI	5.0 × 10^2^	Al_2_O_3_	0.20	NI	20	NI	2.0	NI	NI	NI	NI	[13]
fermentation soy sauce	NI	3.2 × 10^3^	Al_2_O_3_; ZrO_2_	0.20; 0.50; 0.80	2.32 × 10^11^; 1.88 × 10^11 3^; 1.80 × 10^11 3^	22 ± 3	0.05– 0.20	0.3– 0.6	2.0% NaOH; 0.15 M HNO_3_	40 ± 3	NI	NI	[14]
fermentation broth	*Bacillus coagulans* (A20; A369; A107; A59)	NI	ZrO_2_–TiO_2_	0.20	NI	NI	0.15	NI	NI	NI	NI	NI	[15]
fermentation broth	*Lactobacillus helveticus* CNRZ 303	1.0 × 10^9^–5.0 × 10^9^	Al_2_O_3_	0.20	(1.70 ± 0.20) × 10^11^	43 ± 1	0.01– 0.29	6.0 ± 1.0	NaClO; 0.03 M HNO_3_	50	40	7.0	[16]
fermentation broth	*Lactobacillus delbrueckii* spp. *lactis* (Ezal LB 120)	NI	Al_2_O_3_, TiO_2_	0.10	NI	48	0.15; 0.20	3.0; 4.0	5 g/L Ultrasil 25 F; 10 g/L HNO_3_	50; 85	NI	NI	[17]
fermentation broth	*Saccharopolyspora* *erythraea* CA340	NI	NI	0.20	NI	21	0.01– 0.08	0.66	5% hypochlorite; 5% Redphos Special	50	60	NI	[18]
fermentation broth	*Sinorhizobium meliloti* M5N1	9.0 × 10^8^	NI	0.50	NI	30	0.04; 0.10	NI	chlorine solution	70	60	NI	[19]
fermentation broth	*Bacillus velezensis*	2.2 10^9^–2.3 × 10^9^	NI	0.20	NI	25	0.02; 0.06; 0.10	0.4; 0.9; 1.3	acid–base cleaning sequence	NI	NI	NI	[20]
fermentation broth	*Lactobacillus delbrueckii* ssp. *lactis*	NI	Al_2_O_3_, TiO_2_	0.10; 0.80	3.27 × 10^11^; 6.00 × 10^10^	44	0.05– 0.20	4.0	5 g/L Ultrasil 25 F; 10 g/L HNO_3_	50; 85	NI	NI	[21]
fermentation broth	*Lactococcus lactis* ssp. lactis ATCC 19 435	NI	Al_2_O_3_	0.20	NI	30	0.03– 0.14	5.3– 10.8	0.2% Ultrasil 10; 0.4% Ultrasil 10; 0.2% Ultrasil 10	50; 50; NI	NI, 60, NI ^4^	NI	[22]
fermentation broth	*Lactobacillus delbrueckii* subsp. *bulgaricus* CFL1	1.3 × 10 ± 1.3 × 10^7^	Al_2_O_3_	0.10	1.74 × 10^12^	NI	0.02– 0.46	0.1– 11.0	1.0% Ultrasil 25F; 0.2% HNO_3_; 1.0% NaOH + 200 mg/L NaClO	45 and 80; 30; 20	10 and 30; 15; 30	NI	[23]
fermentation broth	*Actinobacillus succinogenes* ATCC 55618	NI	NI	0.10	4.90 × 10^12^	30	NI	0.1	1.0% NaOH; 1.5% H_3_PO_4_	NI	NI	NI	[24]
fermentation broth	NI	NI	ZrO_2_	0.05; 0.20; 0.50	1.96 × 10^11 3^	20–40	0.05– 0.20	2.0– 5.0	0.1% NaOH; 0.1 mol/L C₂H₂O₄	40 ± 3	30; 20	NI	[25]

NI—no information; ^1^ flow rate equal to 0.5 dm^3^/s; ^2^ flow rate between 6 and 10 dm^3^/min; ^3^ data obtained from a graph; ^4^ until the day prior to the next use.
